# The effect of air pollution on catastrophic health expenditure among middle-aged and older adults in China

**DOI:** 10.1371/journal.pone.0347317

**Published:** 2026-04-21

**Authors:** Huan He, Xuanhan Li, Lanxi Peng

**Affiliations:** School of Public Administration, Southwestern University of Finance and Economics, Chengdu, Sichuan, China; Planetary Health Research Center, NEPAL

## Abstract

**Background:**

Catastrophic health expenditure (CHE) reflects the financial protection level towards the universal health coverage target. Air pollution is a major contributor to the economic burden of diseases. However, the relationship between air pollution and CHE remains largely unexplored. This study aims to investigate the effect of air pollution on CHE among middle-aged and older adults in China, and whether this effect is ameliorated by social health insurance.

**Methods:**

Using four waves of the China Health and Retirement Longitudinal Study data matched with PM_2.5_ and meteorological data, we included 24004 participants aged 45 and above in the analysis. CHE was defined as out-of-pocket health expenditure exceeding 40% of non-food expenditure. We employed a random effects logit model and instrumental variable estimation to examine the effect of annual average PM_2.5_ concentration on CHE, and analyzed mechanisms and subgroup heterogeneity. Besides, the interaction effects of social health insurance types and PM_2.5_ on CHE were tested.

**Results:**

PM_2.5_ was significantly associated with an increased likelihood of CHE among middle-aged and older adults in China. Increased exposure to PM_2.5_ may exacerbate CHE by elevating the direct costs of diseases and diminishing labor supply. Heterogeneity analysis indicated that those living in urban areas, aged above 65 years, with low educational attainment, and chronic conditions are more susceptible to PM_2.5_ than their counterparts. Further analysis revealed that only enrollment in the Urban and Rural Residents Medical Insurance, a basic health insurance with low premiums, mitigated the effect of PM_2.5_ on CHE among social health insurance types.

**Conclusions:**

This study provides novel evidence that improved air quality lowers the likelihood of CHE, potentially through reduced disease-related direct costs and increased labor supply. These findings underscore the necessity of air pollution regulations and offer valuable insights for developing effective strategies to prevent air pollution and illness-related poverty.

## Introduction

Catastrophic health expenditure (CHE) refers to a situation in which out-of-pocket healthcare spending exceeds a certain percentage of the household’s capacity to pay [1–3], reflecting whether a household experiences financial hardship due to medical expenditure. CHE is an established indicator used to monitor global progress towards the financial protection dimension of the universal health coverage target prioritized in the Sustainable Development Goals (SDGs) [1]. Globally, approximately 1 billion people experienced financial hardship due to out-of-pocket healthcare spending in 2019, and the portion of the population incurring CHE increased continuously from 9.6% in 2000 to 13.5% in 2019 [4]. CHE is more prevalent among households with older members [5–8], and in low- and middle-income countries (LMICs) [2,9]. As one of the largest LMICs, China is experiencing rapid population aging, with the proportion of people aged 65 and above rising from 10.5% in 2015 to 15.4% in 2023 [10], which raises concerns about the CHE problem among older people. However, previous studies on identifying determinants of CHE have predominantly focused on health system factors (i.e., medical insurance) and individual factors (i.e., diseases and socioeconomic status) [11–13], ignoring possible environmental factors, especially for air pollution, which is a leading cause of economic disease burden [14,15].

Air pollution is one of the most pressing global public health threats of the 21st century. Beyond its well-established direct health effects—such as respiratory and cardiovascular diseases, cancer, and impaired cognitive function [16–18]—air pollution exacerbates healthcare utilization and financial burdens, particularly in LMICs undergoing rapid industrialization [19,20]. Among air pollutants, exposure to particulate matter smaller than 2.5 micrometers (PM_2.5_) is the leading contributor to disability-adjusted life-years globally, according to population attributable fraction estimates from the Global Burden of Disease Study 2021 [21]. Air pollution may increase residents’ medical spending, imposing considerable burdens on individuals and households [22–27]. This burden is epitomized by CHE, which may be disastrous for vulnerable families and perpetuates intergenerational transmission of health inequalities. However, the association between air pollution and CHE is understudied, especially compared to the prior focus on health outcomes. Only one study empirically explored the relationship between air pollution and CHE at the aggregated city level by a geographical detector model and reported a positive association [28]. The effect of air pollution on CHE at the individual level and its underlying mechanisms has not been examined, leaving a critical gap in understanding how air quality affects family financial risks. This may limit policymakers’ ability to design effective interventions that reach both health and economic welfare targets at the same time.

Exploring the mechanisms between air pollution and CHE is essential for preventing the occurrence of CHE. Air pollution may affect CHE through two possible mechanisms: direct costs of diseases and labor supply. On the one hand, air pollution positively impacts out-of-pocket medical expenditure for patients [25,26]. The increased out-of-pocket medical spending caused by air pollution may exceed the threshold of the household’s capacity to pay, which raises the likelihood of CHE. On the other hand, air pollution may impair labor supply through health losses (e.g., requesting medical leave) and avoidance behaviors (e.g., reducing outdoor working times). Empirical evidence showed that air pollution could reduce working times [29–32], labor participation rates [31], and workers’ productivity [33,34]. These labor supply reductions decreased household income and the capacity to pay, further evaluating the likelihood of CHE.

Moreover, social health insurance is important in reducing residents’ medical burden and preventing people from falling into poverty due to illness [35,36]. However, the moderating effect of social health insurance on the effect of air pollution on CHE has not been studied. China has almost achieved universal health insurance coverage, with over 95% of the population enrolled in two types of basic social medical insurance: Urban Employee Basic Medical Insurance (UEBMI) and Urban and Rural Residents Medical Insurance (URRMI) [37]. A recent study found that, on average, enrollment in social health insurance plans did not reduce medical expenditure caused by air pollution among low-income Chinese residents [25], which did not further explore the differential effects of varied basic social medical insurance types. There are significant differences in premiums, reimbursement ratios, and enrollee characteristics across basic social medical insurance types. Identifying the impacts of basic social medical insurance types on the association between air pollution and CHE is important for evaluating their financial protection effects.

This study aims to investigate the relationship between air pollution and CHE. To address this objective, we utilized data from 2011, 2013, 2015, and 2018 waves of the China Health and Retirement Longitudinal Study (CHARLS). The CHARLS data were matched with satellite-based air pollution and meteorological data by city of residence and interview month for each interviewee. We employed a random effects logit model and instrumental variable (IV) estimation to examine the effect of air pollution on CHE, and implemented a series of robustness tests. We explored the mechanisms through which air pollution affects CHE, focusing on the roles of direct costs of diseases and labor supply. Subgroup analyses were employed to examine the heterogeneous effects of air pollution by region, age, education, and chronic disease. Furthermore, we tested the interaction effects between different basic social medical insurance types and PM_2.5_ to identify the moderating effect of social health insurance.

## Materials and methods

### Data

We used four waves of the CHARLS administered in 2011, 2013, 2015, and 2018 to examine the association between PM_2.5_ and CHE. CHARLS is an interview-based nationally representative survey targeting populations aged 45 years and above in China [38]. The survey adopted a stratified multi-stage probability proportional to size random sampling strategy. CHARLS covers approximately 28 provinces, 150 cities (including prefecture-level and county-level cities), and 450 villages. The Biomedical Ethics Review Committee of Peking University approved the procedures for the CHARLS data collection (IRB00001052–11015). All participants signed and marked (if illiterate) the informed consent. The datasets are available in the CHARLS repository: https://charls.charlsdata.com/pages/data/111/zh-cn.html. The CHARLS questionnaires collected data on demographics, medical expenditure, socioeconomic status, health conditions, and medical insurance. Importantly, CHARLS provides residential city codes, enabling us to merge individual-level data with city-level PM_2.5_ and meteorological data. Participants aged 45 years and older were included in this study. Additionally, we excluded observations with: (1) missing information on out-of-pocket medical expenditure and household expenditures; (2) zero values for household food or nonfood expenditure; (3) missing information on covariates. S1 Fig shows the flowchart of the sample inclusion process. We construct the unbalanced data with a total of 65797 observations from the 24004 participants nested within 125 cities.

PM_2.5_ concentration data were sourced from the Atmospheric Composition Analysis Group (ACAG). The ACAG developed and applied a methodology for PM_2.5_ estimates, which combines satellite retrievals of aerosol optical depth, chemical transport modelling, and ground-based measurements [39]. This study utilized the version V5.GL.03 dataset, which provides gridded monthly PM_2.5_ concentration data at a spatial resolution of 0.01° × 0.01°, available at: https://sites.wustl.edu/acag/datasets/surface-pm2-5/.

Meteorological data, including wind speed, temperature, precipitation, relative humidity, and air pressure, were obtained from ERA5. ERA5 is the fifth-generation atmospheric reanalysis dataset for the global climate and weather developed by the European Centre for Medium-Range Weather Forecasts (ECMWF). ERA5 provides estimates at a spatial resolution of 0.25° × 0.25°, available at: https://cds.climate.copernicus.eu/datasets/reanalysis-era5-single-levels?tab=overview.

### Variables

CHE was defined as out-of-pocket health spending exceeding 40% of a household’s capacity to pay, a threshold widely adopted in prior studies [1–3]. CHE was a binary variable, indicating whether the participant experienced CHE. Household’s capacity to pay was operationalized as total household expenditure minus food expenditure [3,40]. The measurement of total household expenditure included spending on food and non-food items, including dining out, clothing, travelling, entertainment, communications, and other items. Household annual out-of-pocket healthcare expenditure was calculated by summing up the costs of participants and their spouses on inpatient and outpatient care in the past year [40]. CHARLS collected self-reported out-of-pocket payments for inpatient care during the past year, but for outpatient care over the last month. Annual out-of-pocket expenditure on outpatient care was calculated as the monthly outpatient expenditure multiplied by 12, following established practices [40–44]. All expenses were adjusted using the consumer price index. We further examined the robustness of the findings by using alternative CHE thresholds.

In the mechanism analysis, direct costs of diseases and labor supply were regarded as intermediate variables. The participants’ direct costs of diseases were measured by two indicators: outpatient and inpatient out-of-pocket medical expenditures. Labor supply was assessed using working hours and health-related work absenteeism. The CHARLS questionnaire inquired about working hours and health-related work absenteeism for workers (non-retired), including four working types: agricultural self-employment, non-agricultural employment, side job (did not ask about health-related work absenteeism), and non-agricultural self-employment and assisting with family business activities. Working hours referred to the number of hours worked in the last year, calculated based on self-reported working months, average working days per week, and average working hours per day. Health-related work absenteeism was the number of days off work due to health problems in the previous year.

The annual average PM_2.5_ concentration was used to measure air pollution, as PM_2.5_ was the primary air pollutant in China during the survey period. In 2017, over 70% of the severe air pollution days in China were attributable to PM_2.5_ pollution [45]. We calculated city-level average PM_2.5_ concentration over the 12 months preceding the interview date for each participant. Following the relevant literature, we employed wind speed as an IV for PM_2.5_ concentration [31,46]. The annual average wind speed was calculated using the same method as for the PM_2.5_ data.

Referring to the previous studies, our analysis included individual and weather covariates [5,47,48]. Individual covariates consisted of age, male, married, urban, schooling years, per capita household expenditure, drinking, smoking, household size, cooking fuel, activities of daily living difficulty, chronic disease, and medical insurance. The types of medical insurance included Urban Employee Basic Medical Insurance (UEBMI), Urban Resident Basic Medical Insurance (URBMI), New Rural Cooperative Medical Scheme (NRCMS), Urban and Rural Residents Medical Insurance (URRMI), other medical insurance (including government medical assistance and commercial medical insurance), and uninsured.

Weather covariates included temperature bins, precipitation, relative humidity, and air pressure. We defined the exposure window for weather covariates as the 12 months preceding the survey. Furthermore, accounting for the potential non-linear effect of temperature on health expenditure [49,50], we first constructed temperature bins based on daily average temperature: (below 0 °C), [0 °C-10 °C), [10 °C-20 °C), [20 °C-30 °C), [30 °C above), and then summed the number of days in each temperature bins in the 12-month exposure window as the indicator for temperature.

### Empirical strategy

We employed a logit model for panel data to examine the association between PM_2.5_ and CHE. The Hausman test compared estimates from random and fixed effects logit models. Hausman test results supported using the random effects logit model as our benchmark model. The random effects logit model can be constructed as follows:


$$\begin{array}{c} P({CHE}_{it}=\text{1})=\Lambda (\alpha +\beta {PM}_{ct}+\gamma \overset{\prime }{\underset{it}{X}}+\theta \overset{\prime }{\underset{ct}{W}}+\mu_{i}+\omega_{t}+\varepsilon_{it}) \end{array}$$

(1)

where *i*, *c*, and *t* index individual, city, and time. ${CHE}_{it}$ is a dummy that equals one if individual *i* incurs CHE and zero otherwise. *PM* is the annual average PM_2.5_ concentration in city *c*. $\overset{\prime }{\underset{it}{X}}$ represents individual covariates, including age, male, married, urban, schooling years, per capita household expenditure, drinking, smoking, household size, cooking fuel, activities of daily living difficulty, chronic disease, and medical insurance. $\overset{\prime }{\underset{ct}{W}}$ represents weather covariates, including temperature bins, precipitation, relative humidity, and air pressure. β, γ, and θ are regression coefficients. $\mu_{i}$ is the individual random effect, and $\omega_{t}$ is the time effect. Λ is the logistic distribution function.

We clustered the standard errors at the household level to address the autocorrelation in CHE for the same households. Besides, a two-stage IV estimation was employed to alleviate potential endogeneity bias.

Based on the random effects logit model estimates, we calculated the average marginal effect of each variable on the probability of CHE, which represents the impact of a unit change in the explanatory variable on the likelihood of CHE. The average marginal effect of the explanatory variable is the derivative of the predicted probability for the explanatory variable, and its standard error is estimated using the delta method. Previous literature recommended the average marginal effect when exploring how a change in a continuous independent variable affects the probability of the dependent variable [51]. Furthermore, a two-sided *p*-value < 0.1 was considered statistically significant.

## Results

### Descriptive statistics

Table 1 reports the summary statistics of the sample. In the study sample, the occurrence of CHE was 18%. The means of outpatient and inpatient out-of-pocket medical expenditures were 161.71 and 951.21 CNY, respectively. The average working time was 1689.79 hours per year, and the annual average health-related work absenteeism was 14.55 days. During the study period, the annual average PM_2.5_ concentration was 46.18 μg/m^3^. The mean age of the sample was 60.2 years. 49% of the sample were male, 88% were married, and 40% were urban residents. The average schooling years was 5.45 years. The average per capita household expenditure was 13962.68 CNY. 43% and 42% of the sample were drinking and smoking, respectively. The average household size was 3.26 members. Regarding cooking fuel, 41% used solid fuels, approximately 57% of the sample used non‑solid fuels, and 1% reported using other fuels or did not cook. For health characteristics, 5% of the sample needed help with their daily activities, and the majority (72%) had chronic diseases.

**Table 1:** Summary statistics of the sample.

Variable	Definition	Mean (SD) or %
**Catastrophic health expenditure**	Yes = 1, no = 0	0.18 (0.38)
**Outpatient out-of-pocket medical expenditure**	Natural logarithm of outpatient out-of-pocket payments during the past year	0.96 (2.18)
**Inpatient out-of-pocket medical expenditure**	Natural logarithm of inpatient out-of-pocket payments during the past month	0.95 (2.63)
**Working hours** ^**a**^	Natural logarithm of working hours	6.89 (1.41)
**Health-related work absenteeism** ^**b**^	Natural logarithm of days off work due to health problems	1.00 (1.60)
**PM**_**2.5**_ **(μg/m**^**3**^)	Annual average PM_2.5_ concentration	46.18 (16.43)
**Wind Speed (m/s)**	Annual average wind speed	2.51 (0.61)
**Age**	The participant’s age	60.20 (9.67)
**Male**	Male = 1, female = 0	0.49 (0.50)
**Married**	Married = 1, unmarried = 0	0.88 (0.32)
**Urban**	Residence area: urban = 1, rural = 0	0.40 (0.49)
**Schooling years**	Convert education level into schooling years, ranging from 0 (illiterate) to 23 (doctoral degree)	5.45 (4.18)
**Per capita household expenditure**	Natural logarithm of per capita household expenditure	9.04 (0.95)
**Drinking**	Yes = 1, no = 0	0.43 (0.49)
**Smoking**	Yes = 1, no = 0	0.42 (0.49)
**Household size**	Number of family members living together	3.26 (1.63)
**Cooking fuel**	Solid fuels (coal or crop residue/wood burning)	41.43%
	Non-solid fuels (natural gas, marsh gas, liquefied petroleum gas, or electric)	57.45%
	Others (other fuels or do not cook)	1.11%
**Activities of daily living difficulty**	Yes = 1, no = 0	0.05 (0.22)
**Chronic disease**	Yes = 1, no = 0	0.72 (0.45)
**Medical insurance**	UEBMI	11.26%
	URBMI	4.22%
	NRCMS	67.44%
	URRMI	4.20%
	Other medical insurance	7.20%
	Uninsured	5.68%
**Temperature (°C)** ^**c**^	Annual average temperature	14.19 (5.33)
**Precipitation (mm)**	Annual cumulative precipitation	1082.68 (458.21)
**Relative humidity (%)**	Annual average relative humidity	60.69 (7.09)
**Air pressure (hPa)**	Annual average air pressure	953.01 (73.26)

Notes: Total observations = 65797. ^a^ The number of observations of working hours is 41437. ^b^ The number of observations of health-related work absenteeism is 43127. ^c^ For interpretability, we present the annual average temperature here instead of the number of days in temperature bins. UEBMI, Urban Employee Basic Medical Insurance; URBMI, Urban Resident Basic Medical Insurance; NRCMS, New Rural Cooperative Medical Scheme; URRMI, Urban and Rural Residents Medical Insurance.

### Benchmark model results

Table 2 reports the estimated association between PM_2.5_ and CHE, with columns (1) to (3) showing the results by adding covariates. The results reveal a statistically positive relationship between PM_2.5_ and CHE. The estimates, controlling for all covariates, in column (3) show that a 10 μg/m^3^ increase in annual average PM_2.5_ concentration was associated with a 0.5% increase in the likelihood of CHE.

**Table 2:** The effect of PM_2.5_ on catastrophic health expenditure.

Variables	Catastrophic health expenditure		
	(1)	(2)	(3)
**PM**_**2.5**_ **(10 μg/m**^**3**^)	0.006^**^ (0.002)	0.005^**^ (0.002)	0.005^**^ (0.002)
**Age**		0.002^***^ (0.0002)	0.002^***^ (0.0002)
**Male**		−0.0004 (0.004)	0.0003 (0.004)
**Married**		0.115^***^ (0.006)	0.114^***^ (0.006)
**Urban**		−0.019^***^ (0.005)	−0.016^***^ (0.005)
**Schooling years**		−0.002^***^ (0.001)	−0.002^***^ (0.001)
**Per capita household expenditure**		−0.018^***^ (0.002)	−0.017^***^ (0.002)
**Drinking**		−0.009^**^ (0.004)	−0.009^**^ (0.004)
**Smoking**		0.004 (0.005)	0.004 (0.005)
**Household size**		−0.016^***^ (0.001)	−0.017^***^ (0.001)
**Cooking fuel (Ref = Non-solid fuels)**			
**Solid fuels**		0.023^***^ (0.005)	0.022^***^ (0.005)
**Others**		−0.019 (0.020)	−0.021 (0.020)
**Activities of daily living difficulty**		0.087^***^ (0.006)	0.087^***^ (0.006)
**Chronic disease**		0.084^***^ (0.004)	0.083^***^ (0.004)
**Medical insurance (Ref = No insurance)**			
**UEBMI**			0.019^*^ (0.010)
**URBMI**			0.045^***^ (0.012)
**NRCMS**			0.037^***^ (0.008)
**URRMI**			0.024^*^ (0.012)
**Other medical insurance**			0.023^**^ (0.010)
**Weather covariates**	Yes	Yes	Yes
**Year dummies**	Yes	Yes	Yes
**Individual random effects**	Yes	Yes	Yes
**Observations**	65797	65797	65797

Notes: The reported coefficients are the estimated marginal effects. Robust standard errors in parentheses are clustered at the household level. UEBMI, Urban Employee Basic Medical Insurance; URBMI, Urban Resident Basic Medical Insurance; NRCMS, New Rural Cooperative Medical Scheme; URRMI, Urban and Rural Residents Medical Insurance. ^*^p < 0.1, ^**^p < 0.05, ^***^p < 0.01.

### Instrumental variable estimation

Although the benchmark model controlled for individual and time effects and included numerous covariates, some unobserved confounders correlated with air pollution and CHE might remain. IV estimation was employed to address this problem, using a commonly used IV for air pollution, i.e., wind speed [31,46]. Wind speed is negatively associated with PM_2.5_, and there is no evidence that wind speed directly impacts residents’ medical expenditure and CHE.

IV estimation is typically implemented using a two-stage regression. Specifically, in the first stage, we regressed PM_2.5_ concentration on wind speed to generate the predicted PM_2.5_. In the second stage, we estimated the effect of predicted PM_2.5_ on CHE. Due to the difficulty in accommodating non‐linear IV estimation with panel data, we adopted a linear probability model (LPM) in the second-stage IV regression instead of the logit model (used in the benchmark model). Additionally, we used LPM to estimate the effect of PM_2.5_ on CHE to demonstrate the validity of LPM estimation. Column (1) in Table 3 presents the LPM results. The LPM results show that the directions and magnitudes of the effects were similar to the logit model results. Column (2) reports the first-stage results of the IV estimation, demonstrating a significantly negative association between wind speed and PM_2.5._ The F statistic was greater than 10, suggesting that the hypothesis of weak IV was rejected. Column (3) presents the second stage results of the IV estimation, showing that PM_2.5_ increased the likelihood of CHE.

**Table 3:** Results of linear probability model and instrumental variable estimation.

Variables	Catastrophic health expenditure		
	LPM	IV	
	(1)	(2) First stage	(3) Second stage
PM_2.5_ (10 μg/m^3^)	0.005^**^ (0.002)		0.017^***^ (0.005)
Wind speed		−0.994^***^ (0.181)	
Individual covariates	Yes	Yes	Yes
Weather covariates	Yes	Yes	Yes
Year dummies	Yes	Yes	Yes
Individual random effects	Yes	Yes	Yes
F Statistic		72379.95	
Observations	65797	65797	65797

Notes: Robust standard errors in parentheses are clustered at the household level. LPM, linear probability model; IV, instrumental variable. ^*^p < 0.1, ^**^p < 0.05, ^***^p < 0.01.

### Robustness tests

A variety of robustness checks were conducted to confirm our findings. First, we used different CHE thresholds proposed by the World Health Organization (WHO) and the World Bank [1]. Specifically, CHE was redefined using three criteria: (1) 10% of total household expenditure, (2) 25% of total household expenditure, or (3) 25% of household non-food expenditure. The results are presented in Table 4. The relationship between PM_2.5_ and CHE remained significantly positive regardless of different thresholds. The results were consistent with the benchmark results. The results in Table 4 demonstrate that our findings are not sensitive to the CHE thresholds.

**Table 4:** Estimates at different thresholds for catastrophic health expenditure.

Variables	Catastrophic health expenditure		
	(1)	(2)	(3)
	10% of total household expenditure	25% of total household expenditure	25% of household non-food expenditure
**PM**_**2.5**_ **(10 μg/m**^**3**^)	0.006^**^ (0.003)	0.004^**^ (0.002)	0.007^***^ (0.003)
**Individual covariates**	Yes	Yes	Yes
**Weather covariates**	Yes	Yes	Yes
**Year dummies**	Yes	Yes	Yes
**Individual random effects**	Yes	Yes	Yes
**Mean of CHE**	0.238	0.148	0.230
**Observations**	65797	65797	65797

Notes: The reported coefficients are the estimated marginal effects. Robust standard errors in parentheses are clustered at the household level. In column (1), catastrophic health expenditure is redefined as 10% of total household expenditure; in column (2), catastrophic health expenditure is redefined as 25% of total household expenditure; in column (3), catastrophic health expenditure is redefined as 25% of household non-food expenditure. CHE, catastrophic health expenditure. ^*^p < 0.1, ^**^p < 0.05, ^***^p < 0.01.

Second, we tested the robustness to outliers by winsorizing out-of-pocket healthcare expenditures at the top 1%, 2%, and 3% of the distribution. Table 5 presents the results for winsorizing the out-of-pocket health expenditures. The results remained consistent with our benchmark results, suggesting that our main finding was robust.

**Table 5:** Estimates after winsorizing the out-of-pocket health expenditure.

Variables	Catastrophic health expenditure		
	(1)	(2)	(3)
	Winsorize the top 1%	Winsorize the top 2%	Winsorize the top 3%
**PM**_**2.5**_ **(10 μg/m**^**3**^)	0.005^**^ (0.002)	0.005^*^ (0.002)	0.005^**^ (0.002)
**Individual covariates**	Yes	Yes	Yes
**Weather covariates**	Yes	Yes	Yes
**Year dummies**	Yes	Yes	Yes
**Individual random effects**	Yes	Yes	Yes
**Mean of CHE**	0.180	0.179	0.177
**Observations**	65797	65797	65797

Notes: The reported coefficients are the estimated marginal effects. Robust standard errors in parentheses are clustered at the household level. CHE, catastrophic health expenditure. ^*^p < 0.1, ^**^p < 0.05, ^***^p < 0.01.

Third, to account for potential confounding by the regional economic development and medical resources, we added additional city covariates into the benchmark model, including per capita GDP and doctors per thousand inhabitants collected from the *China City Statistical Yearbook*. These variables were not included in the benchmark model due to concerns of potential endogeneity with air pollution. As shown in Table 6, the result consistently supports that air pollution was positively associated with CHE, though the magnitude of effect was slightly smaller than that of the benchmark model. The result indicates that our main findings are still robust after adjusting for additional city covariates.

**Table 6:** Estimates after adding city covariates.

Variables	Catastrophic health expenditure
**PM**_**2.5**_ **(10 μg/m**^**3**^)	0.004^*^ (0.002)
**Individual covariates**	Yes
**City covariates**	Yes
**Weather covariates**	Yes
**Year dummies**	Yes
**Individual random effects**	Yes
**Observations**	65797

Notes: The reported coefficients are the estimated marginal effects. Robust standard errors in parentheses are clustered at the household level. ^*^p < 0.1, ^**^p < 0.05, ^***^p < 0.01.

Fourth, although the number of missing covariate data is small (accounting for approximately 1.6% of the analytical sample), this may bias the results to some extent. To examine the influence of missingness in covariates, we coded the missing values as a missing group for categorical covariates to perform full sample analysis. The result presented in Table 7 is similar to our benchmark results, which suggests the benchmark results are not much affected by missing covariate data.

**Table 7:** Estimates after including observations with missing data for categorical covariates.

Variables	Catastrophic health expenditure
**PM**_**2.5**_ **(10 μg/m**^**3**^)	0.003^*^ (0.001)
**Individual covariates**	Yes
**Weather covariates**	Yes
**Year dummies**	Yes
**Individual random effects**	Yes
**Observations**	66865

Notes: The reported coefficients are the estimated marginal effects. Robust standard errors in parentheses are clustered at the household level. ^*^p < 0.1, ^**^p < 0.05, ^***^p < 0.01.

### Mechanism analysis

We investigated two important mechanisms underlying the relationship between air pollution and CHE: direct costs of diseases and labor supply. Given that the direct costs of diseases and labor supply measurements exhibited a large proportion of zero values, we used a Tobit model to estimate the effect of air pollution on these variables [52]. The random effects Tobit model does not allow for cluster robust standard errors. Thus, we used bootstrapped standard errors for statistical examination [53].

First, we tested the direct costs of diseases channel by analyzing participants’ out-of-pocket medical expenditure. Specifically, we separately examined the effect of PM_2.5_ on outpatient and inpatient out-of-pocket medical expenditures. As shown in Table 8, outpatient and inpatient out-of-pocket medical expenditures increased by 1.8% and 7%, respectively, with a 10 μg/m^3^ rise in PM_2.5_ concentration.

**Table 8:** The effect of PM_2.5_ on the direct costs of diseases.

Variables	Direct costs of diseases	
	(1)	(2)
	Outpatient out-of-pocket medical expenditure	Inpatient out-of-pocket medical expenditure
**PM**_**2.5**_ **(10 μg/m**^**3**^)	0.018^*^ (0.010)	0.070^***^ (0.011)
**Individual covariates**	Yes	Yes
**Weather covariates**	Yes	Yes
**Year dummies**	Yes	Yes
**Individual random effects**	Yes	Yes
**Observations**	65797	65797

Notes: The reported coefficients are the estimated marginal effects. Bootstrapped standard errors are in parentheses. ^*^p < 0.1, ^**^p < 0.05, ^***^p < 0.01.

Second, we tested the labor supply channel using working hours and health-related work absenteeism. Table 9 presents the effect of PM_2.5_ on labor supply. Column (1) shows that a 10 μg/m^3^ increase in PM_2.5_ concentration was associated with a 1.9% reduction in working hours. Column (2) reports that each 10 μg/m^3^ PM_2.5_ concentration increase was associated with a 3.7% rise in health-related work absenteeism. Given that the effect of air pollution on labor supply may differ across working types, we further examined the above effect using subsamples of different working types to ensure the robustness of our mechanism analysis. S1 Table shows that the results for the impact of PM_2.5_ on labor supply across working types are generally robust. PM_2.5_ was significantly negatively associated with the working hours of the last year among the non-agricultural workers. The effects of PM_2.5_ on working hours for the other three working types are negative but not statistically significant. The impact of PM_2.5_ on the health-related work absenteeism was significantly positive for the agricultural self-employed, while this effect was not statistically significant for other working types.

**Table 9:** The effect of PM_2.5_ on labor supply.

Variables	Labor supply	
	(1)	(2)
	Working hours	Health-related work absenteeism
**PM**_**2.5**_ **(10 μg/m**^**3**^)	−0.019^**^ (0.008)	0.037^***^ (0.009)
**Individual covariates**	Yes	Yes
**Weather covariates**	Yes	Yes
**Year dummies**	Yes	Yes
**Individual random effects**	Yes	Yes
**Observations**	41437	43127

Notes: The reported coefficients are the estimated marginal effects. Bootstrapped standard errors are in parentheses. ^*^p < 0.1, ^**^p < 0.05, ^***^p < 0.01.

### Heterogeneity analysis

**Fig 1:**
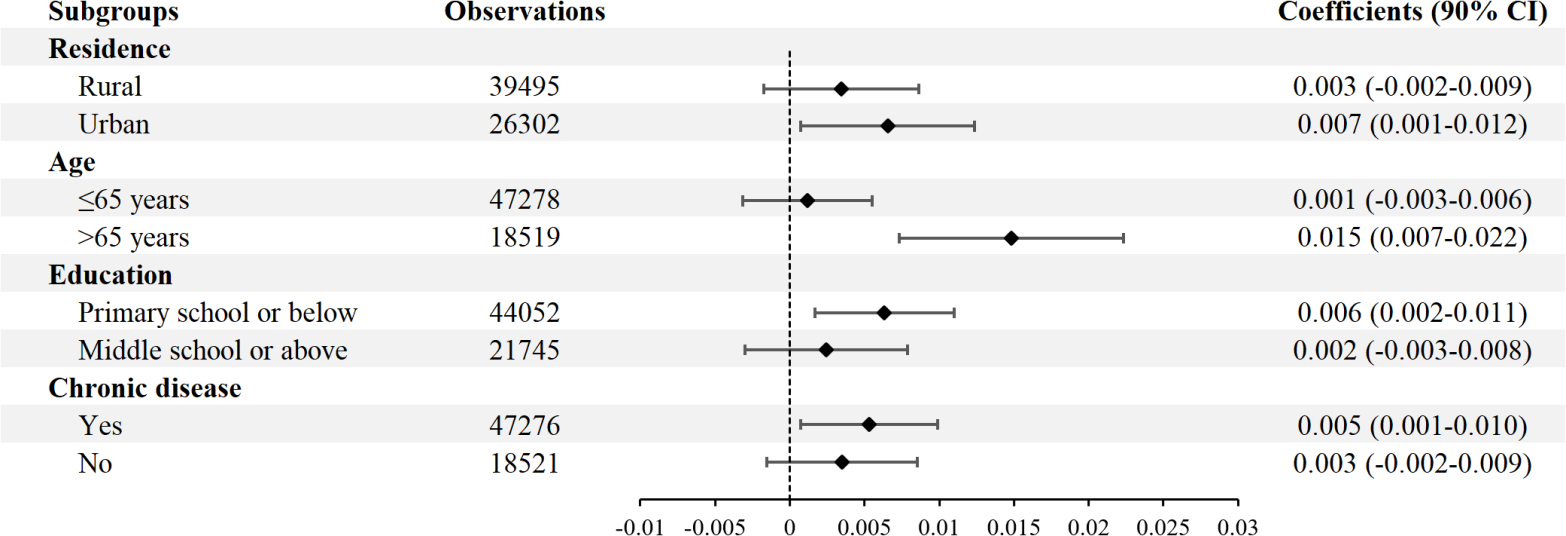
Heterogeneity analysis of PM_2.5_ effects on catastrophic health expenditure. This figure presents subgroup analyses by residence, age, education, and chronic disease. The diamonds represent the estimated marginal effects of PM_2.5_ exposure, and horizontal bars represent the 90% confidence interval. All regressions included individual covariates, weather covariates, year dummies, and individual random effects. Robust standard errors in parentheses are clustered at the household level. CI, Confidence Interval.

We also investigate whether the effect of PM_2.5_ on CHE varies by individual characteristics. Fig 1 shows the results of the heterogeneity analysis. The effect of PM_2.5_ on CHE was positive and statistically significant among urban residents, but it was insignificant among rural residents.

We use a cutoff age of 65 years to separate participants into middle-aged and older adult groups. The findings show that PM_2.5_ was positively and significantly associated with CHE in the older group, whereas no such association was observed in the middle-aged group.

Based on educational attainment, we divided the participants into two groups: individuals with primary school or below educational level and those with middle school or above degrees. PM_2.5_ was positively associated with CHE for individuals with a primary school or below educational degree. In contrast, the association between PM_2.5_ and CHE was much smaller in effect size and insignificant among individuals with middle school or above degrees.

We divided the sample into subgroups according to whether they had chronic conditions. The results show that the association between PM_2.5_ and CHE was positive and significant among those with chronic diseases, while this association is a bit smaller in effect size and statistically insignificant for those without chronic diseases.

### The moderating effect of social health insurance

This study examined whether social health insurance ameliorates the CHE caused by air pollution. During the survey period, China's social health insurance system comprised four schemes: UEBMI, URBMI, NRCMS, and URRMI. UEBMI primarily covers employees, and the remaining three schemes target non-employed residents. Additionally, URRMI was established by consolidating URBMI and NRCMS.

We incorporated interaction terms between PM_2.5_ and different insurance types into the benchmark model. Table 10 presents the results for the moderating effects of social health insurance types on the association between PM_2.5_ and CHE. The results show that the interaction coefficient between PM_2.5_ and URRMI was significantly negative. In contrast, the interaction coefficients between PM_2.5_ and other medical insurance types were negative but statistically insignificant. These findings demonstrate that URRMI plays a significant role in reducing CHE caused by PM_2.5_.

**Table 10:** The moderating effects of social health insurance types.

Variables	Catastrophic health expenditure
**PM**_**2.5**_ **(10 μg/m**^**3**^)	0.010^**^ (0.005)
**PM**_**2.5**_ **× No insurance (Ref)**	
**PM**_**2.5**_ **× UEBMI**	−0.003 (0.006)
**PM**_**2.5**_ **× URBMI**	−0.005 (0.007)
**PM**_**2.5**_ **× NRCMS**	−0.006 (0.005)
**PM**_**2.5**_ **× URRMI**	−0.023^***^ (0.008)
**PM**_**2.5**_ **× Other medical insurance**	−0.002 (0.006)
**Individual covariates**	Yes
**Weather covariates**	Yes
**Year dummies**	Yes
**Individual random effects**	Yes
**Observations**	65797

Notes: The reported coefficients are the estimated marginal effects. Robust standard errors in parentheses are clustered at the household level. UEBMI, Urban Employee Basic Medical Insurance; URBMI, Urban Resident Basic Medical Insurance; NRCMS, New Rural Cooperative Medical Scheme; URRMI, Urban and Rural Residents Medical Insurance. ^*^p < 0.1, ^**^p < 0.05, ^***^p < 0.01.

## Discussion

This study examined the effect of PM_2.5_ on CHE among middle-aged and older adults in China, utilizing four waves of the CHARLS data. Our findings demonstrated that PM_2.5_ significantly increases the likelihood of CHE. Mechanism analysis showed that PM_2.5_ was significantly associated with elevated direct costs of diseases and diminished labor supply. Heterogeneous results revealed that PM_2.5_ had a greater impact on urban residents, older adults, low-education individuals, and patients with chronic diseases than their counterparts. Furthermore, we found that among different social health insurance types, only URRMI can effectively mitigate the effect of PM_2.5_ on CHE.

This study offers a new perspective on mitigating CHE by highlighting the role of air pollution. To the best of our knowledge, this is the first study to assess the effect of air pollution on CHE at the individual level. Zhang et al. [28] is the only related study to examine the association between air pollution and CHE at the city level. Our main findings differ from those of Zhang et al. [28] because the research questions addressed are distinct. That study emphasized the interactions between air pollution and other factors, particularly health service factors, and found that air pollution exacerbated the contribution of health service factors to CHE. By contrast, we found that air pollution increased the likelihood of CHE. The IV estimation and various robustness tests support the credibility of the results. Our finding points to the urgent need for air pollution control to alleviate the household economic burden due to illness.

Our mechanism analysis demonstrated that air pollution might affect CHE through the direct costs of diseases and labor supply. We found that air pollution was positively associated with outpatient and inpatient out-of-pocket medical expenditures, which coincides with existing studies [25,26]. Since out-of-pocket medical expenditure serves as the numerator for calculating CHE, its increase directly raises the likelihood of CHE. Moreover, our findings that air pollution reduced working hours and increased health-related work absenteeism among working people align with prior research [29–32]. Severe exposure to air pollution reduces labor supply, thereby decreasing household capacity to pay and increasing the risk of CHE.

We found that the impact of air pollution on CHE varied across subgroups. First, our findings suggested that urban residents were more susceptible to air pollution than rural residents, consistent with prior literature [23]. This disparity likely stems from the air pollution exposure inequality between urban and rural areas. Studies focused on environmental inequality demonstrated that urban areas have higher PM_2.5_ concentrations than rural areas in China [54,55]. This underscores the need for urban air quality improvement, such as expanding urban green space, reducing emissions from mobile sources, and controlling road dust.

Second, our analysis revealed that the effect of air pollution on CHE was greater for the older populations compared to middle-aged adults. A potential explanation could be that an age-related decline in body function may reduce resistance to air pollution. Some studies have shown a negative association between mucociliary clearance and age [56,57], which increases susceptibility to air pollution. This finding suggests that implementing health promotion programs for older adults can foster healthy aging.

Third, similar to previous studies focused on health [58–60], we found that air pollution imposes a greater economic burden on less educated groups than their counterparts. This is probably due to limited knowledge of pollution-related health risks and constrained economic resources among low-education groups [61,62]. By contrast, higher education groups may adopt protective measures, including purchasing masks or air purifiers to mitigate the adverse effects of air pollution [63].

Fourth, we found that individuals with chronic diseases are more vulnerable to the hazard of air pollution than their counterparts. Previous studies also demonstrated that chronic diseases, such as cardiovascular diseases, diabetes, and respiratory diseases, may exacerbate vulnerability to air pollution through endothelial cell dysfunction and inflammatory mechanisms [64–66]. Our finding suggests that patients with chronic diseases may need more medical financial assistance.

In further exploration of the buffering effect of social health insurance, we found that only URRMI can ameliorate CHE caused by PM_2.5_. Other types of basic social medical insurance might partially buffer the adverse effects of air pollution, but these buffering effects were not statistically significant. These findings contrast with the limited previous research, which suggests that basic social medical insurance does not effectively reduce the effect of air pollution on residents’ medical expenditures [25]. However, that study did not explore the different effects of various basic social medical insurance. Given the differences in reimbursement ratios and enrollee characteristics across basic medical insurance types, their buffering effects may also differ. Specifically, URRMI was formed through the merger of URBMI and NRCMS. Compared with the two other resident medical insurances plans (URBMI and NRCMS), URRMI has a higher reimbursement ratio [67,68], which may reduce the out-of-pocket medical expenditure and the effect of PM_2.5_ on CHE. Surprisingly, despite having a higher reimbursement rate than URRMI, UEBMI fails to mitigate the impact of PM_2.5_ on CHE effectively. This could be attributed to the UEBMI enrollees having more economic resources, and they may prefer to pay more out-of-pocket expenses for better medical services [69–71]. Additionally, in line with existing literature [40,47], we found that individuals with social health insurance may have a higher occurrence of CHE than uninsured individuals, which could be related to moral hazard and adverse selection. Our findings suggest that the government should encourage uninsured residents to enroll in URRMI, a basic health insurance with low premiums that may prevent CHE due to air pollution.

However, this study has several limitations. First, household non-food expenditures and out-of-pocket medical expenditures are self-reported by participants, which may be subject to recall bias. Second, exposure misclassification is inevitable due to city-level PM_2.5_ concentration as a proxy for personal exposure. This misclassification may lead to an underestimation of associations [72]. Third, our findings are subject to limitations related to sample selection. Our analysis is restricted to middle-aged and older adults, and the effects of PM_2.5_ on CHE may be smaller for younger populations who are typically less vulnerable to air pollution. Moreover, within our longitudinal sample, healthy survivor bias could lead to an underestimation of these adverse effects if the most vulnerable individuals were disproportionately lost to follow-up. Fourth, this study excluded those who reported zero values in food or nonfood expenditure due to the infeasibility of calculating CHE. Comparison of sample characteristics shows that the excluded subsample was older and had fewer schooling years than the analytical sample (S2 Table), which indicates there might be an underestimation of the effect of PM_2.5_ on CHE in our study. Fifth, we do not control for potential confounders with data availability and quality concerns in the data source, such as distance to roads and heating fuel. Future studies could more precisely isolate the effect of ambient PM_2.5_ by addressing these data gaps.

## Conclusion

This study advances a comprehensive understanding of the relationship between air pollution and CHE through mechanism analysis, heterogeneity analysis, and assessment of the moderating effect of the social health insurance system. This study provides novel evidence that improved air quality lowers the likelihood of CHE, potentially through reduced disease-related direct costs and increased labor supply. This study suggests the following strategies alleviate the adverse effects: enhancing the urban atmospheric environment, implementing targeted health promotion for older adults, raising pollution hazard awareness among the less educated, and improving medical financial assistance for patients with chronic diseases. Importantly, our findings highlight that URRMI has the most significant buffering effect against air-pollution-induced financial health shocks as compared to other insurance schemes, underscoring the need to reflect upon health insurance policies and the importance of sustaining URRMI in China. These findings offer valuable insights for developing effective strategies to prevent air pollution and illness-related poverty.

## Supporting information

S1 Fig The sample inclusion process. (DOCX)

S1 Table The effect of PM_2.5_ on labor supply across working types. The reported coefficients are the marginal effects. Bootstrapped standard errors are in parentheses. *p < 0.1, **p < 0.05, ***p < 0.01.
(DOCX)

S2 Table The characteristics differences between the analytical sample and the sample with zero expenditure on food or non-food. The independent sample t-test was used here. UEBMI, Urban Employee Basic Medical Insurance; URBMI, Urban Resident Basic Medical Insurance; NRCMS, New Rural Cooperative Medical Scheme; URRMI, Urban and Rural Residents Medical Insurance. *p < 0.1, **p < 0.05, ***p < 0.01.
(DOCX)
