# Supplementary figures and images for: The effect of air pollution on catastrophic health expenditure among middle-aged and older adults in China
Source: PLoS One. 2026 Apr 21;21(4):e0347317. doi: 10.1371/journal.pone.0347317 (PMC13099097; doi:10.1371/journal.pone.0347317)

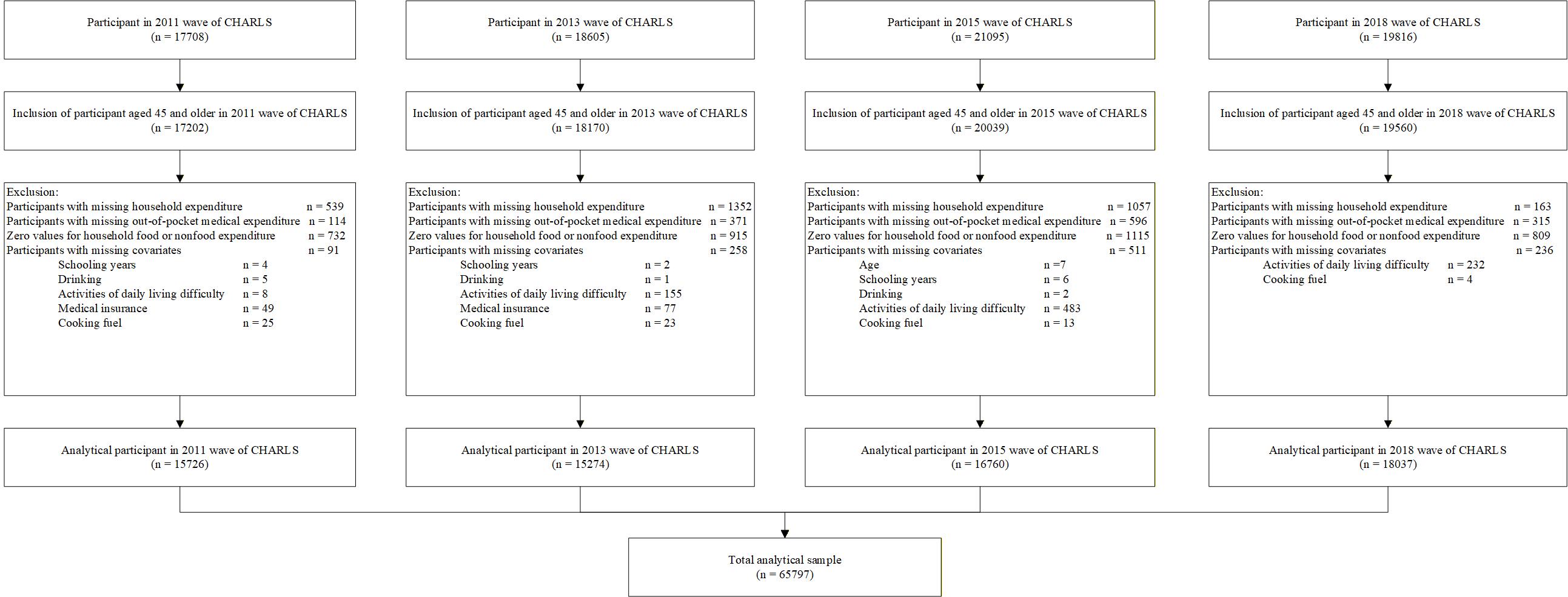


**S1 Fig.** **The** **sample inclusion process.**

Supplement: S1 Fig — (DOCX) [file pone.0347317.s001.docx]
