# Supplementary material for: The effect of air pollution on catastrophic health expenditure among middle-aged and older adults in China
Source: PLoS One. 2026 Apr 21;21(4):e0347317. doi: 10.1371/journal.pone.0347317 (PMC13099097; doi:10.1371/journal.pone.0347317)
Supplement: S1 Table — The reported coefficients are the marginal effects. Bootstrapped standard errors are in parentheses. *p < 0.1, **p < 0.05, ***p < 0.01. (DOCX) [file pone.0347317.s002.docx]

**S1 Table. The effect of PM_2.5_ on labor supply across working types.**

| **Variables** | **Working hours** | | | | **Health-related work absenteeism** | | |
| --- | --- | --- | --- | --- | --- | --- | --- |
|  | **(1)**  **Agricultural**  **self-employment** | **(2)**  **Non-agricultural**  **employment** | **(3)**  **Non-agricultural self-employment and**  **assisting with family**  **business activities** | **(4)**  **Side job** | **(5)**  **Agricultural**  **self-employment** | **(6)**  **Non-agricultural**  **employment** | **(7)**  **Non-agricultural self-employment and**  **assisting with family**  **business activities** |
| **PM_2.5_ (10μg/m^3^)** | -0.009 (0.011) | -0.043^***^ (0.016) | -0.021 (0.030) | -0.075 (0.127) | 0.048^***^ (0.011) | 0.003 (0.009) | -0.010 (0.022) |
| **Individual covariates** | Yes | Yes | Yes | Yes | Yes | Yes | Yes |
| **Weather covariates** | Yes | Yes | Yes | Yes | Yes | Yes | Yes |
| **Year dummies** | Yes | Yes | Yes | Yes | Yes | Yes | Yes |
| **Individual random effects** | Yes | Yes | Yes | Yes | Yes | Yes | Yes |
| **Observations** | 30141 | 12724 | 5620 | 1018 | 32082 | 13128 | 5708 |

Notes: The reported coefficients are the marginal effects. Bootstrapped standard errors are in parentheses. ^*^*p* < 0.1, ^**^*p* < 0.05, ^***^*p* < 0.01.
