# Supplementary material for: The effect of air pollution on catastrophic health expenditure among middle-aged and older adults in China
Source: PLoS One. 2026 Apr 21;21(4):e0347317. doi: 10.1371/journal.pone.0347317 (PMC13099097; doi:10.1371/journal.pone.0347317)
Supplement: S2 Table — The independent sample t-test was used here. UEBMI, Urban Employee Basic Medical Insurance; URBMI, Urban Resident Basic Medical Insurance; NRCMS, New Rural Cooperative Medical Scheme; URRMI, Urban and Rural Residents Medical Insurance. *p < 0.1, **p < 0.05, ***p < 0.01. (DOCX) [file pone.0347317.s003.docx]

**S2 Table. The characteristics differences between the analytical sample and the sample with zero expenditure on food or non-food.**

| **Variable** | **Analytical sample** | | **Sample with zero expenditure on food or non-food** | | ***t-*value** |
| --- | --- | --- | --- | --- | --- |
|  | **Mean** | **Standard deviation** | **Mean** | **Standard deviation** |  |
| PM_2.5_ (μg/m^3^) | 46.175 | 16.427 | 45.723 | 17.090 | 1.585 |
| **Age** | **60.203** | **9.669** | **64.973** | **11.504** | **-28.153^***^** |
| Male | 0.491 | 0.500 | 0.419 | 0.494 | 8.199^***^ |
| Married | 0.885 | 0.319 | 0.717 | 0.451 | 29.617^***^ |
| Urban | 0.400 | 0.490 | 0.380 | 0.485 | 2.351^**^ |
| **Schooling years** | **5.451** | **4.178** | **4.343** | **4.122** | **15.311^***^** |
| Drinking | 0.428 | 0.495 | 0.354 | 0.478 | 8.648^***^ |
| Smoking | 0.421 | 0.494 | 0.352 | 0.478 | 8.126^***^ |
| Household size | 3.260 | 1.634 | 2.883 | 1.644 | 13.326^***^ |
| Cooking fuel |  |  |  |  |  |
| Solid fuels | 0.414 | 0.493 | 0.483 | 0.500 | -8.050^***^ |
| Non-solid fuels | 0.575 | 0.494 | 0.489 | 0.500 | 9.985^***^ |
| Others | 0.011 | 0.105 | 0.028 | 0.165 | -8.928^***^ |
| Activities of daily living difficulty | 0.051 | 0.219 | 0.104 | 0..305 | -13.730^***^ |
| Chronic disease | 0.719 | 0.450 | 0.730 | 0.444 | -1.494 |
| Medical insurance |  |  |  |  |  |
| UEBMI | 0.113 | 0.316 | 0.085 | 0.279 | 5.000^***^ |
| URBMI | 0.042 | 0.201 | 0.043 | 0.203 | -0.266 |
| NRCMS | 0.674 | 0.469 | 0.680 | 0.467 | -0.641 |
| URRMI | 0.042 | 0.201 | 0.040 | 0.196 | 0.572 |
| Other medical insurance | 0.072 | 0.259 | 0.062 | 0.241 | 2.315^**^ |
| Uninsured | 0.057 | 0.231 | 0.090 | 0.287 | -8.221^***^ |
| Observations | 65797 | | 3502 | |  |

Notes: The independent sample t-test was used here. UEBMI, Urban Employee Basic Medical Insurance; URBMI, Urban Resident Basic Medical Insurance; NRCMS, New Rural Cooperative Medical Scheme; URRMI, Urban and Rural Residents Medical Insurance. ^*^*p* < 0.1, ^**^*p* < 0.05, ^***^*p* < 0.01.
